# The prevalence of physical, sexual and mental abuse among adolescents and the association with BMI status

**DOI:** 10.1186/1471-2458-12-840

**Published:** 2012-10-04

**Authors:** Jorien Veldwijk, Karin I Proper, Henriëtte B Hoeven-Mulder, Wanda JE Bemelmans

**Affiliations:** 1 National Institute for Public Health and the Environment, Centre for Prevention and Health Services Research, P.O. Box 1 3720 BA, Bilthoven, The Netherlands; 2 Community Health Service of Gelre IJssel, Apeldoorn, The Netherlands

**Keywords:** Abuse, Weight, Overweight, Obesity, Adolescents

## Abstract

**Background:**

Studies among adults show an association between abuse and Body Mass Index (BMI) status. When an aberrant BMI status as a consequence of abuse is already prevalent in adolescence, early detection and treatment of abuse might prevent these adolescents from developing serious weight problems and other long-term social, emotional and physical problems in adulthood. Therefore, this study investigated the prevalence of physical, sexual and mental abuse among adolescents and examined the association of these abuse subtypes with BMI status.

**Methods:**

In total, data of 51,856 secondary school students aged 13–16 who had completed a questionnaire on health, well-being and lifestyle were used. BMI was classified into four categories, underweight, normal weight, overweight and obesity. Adolescents reported if they had ever been physically, sexually or mentally abused. Crude and adjusted General Estimation Equation (GEE) analyses were performed to investigate the association between abuse subtypes and BMI status. Analyses were adjusted for ethnicity and parental communication, and stratified for gender and educational level.

**Results:**

Eighteen percent of the adolescents reported mental abuse, 7% reported sexual abuse, and 6% reported physical abuse. For underweight, overweight and obese adolescents these percentages were 17%, 25%, and 44%; 7%, 8%, and 16%; and 6%, 8%, 18% respectively. For the entire population, all these subtypes of abuse were associated with being overweight and obese (OR=3.67, 1.79 and 1.50) and all but sexual abuse were associated with underweight (OR=1.21 and 1.12). Stratified analyses showed that physical and sexual abuse were significantly associated with obesity among boys (OR=1.77 and 2.49) and among vocational school students (OR=1.60 and 1.69), and with underweight among girls (OR=1.26 and 0.83).

**Conclusion:**

Mental abuse was reported by almost half of the obese adolescents and associated with underweight, overweight and obesity. Longitudinal analyses are recommended to explore the causality of and the mechanisms explaining this association between abuse and overweight.

## Background

Although there is extensive literature on the association between childhood abuse and eating disorders, less is known about the association between abuse and Body Mass Index (BMI) status in a non-clinical setting. To date, most research focuses on abuse and eating disorders like Anorexia Nervosa and Boulimia Nervosa, that often result in underweight. However, some recent literature suggests that, besides the association with underweight, being abused may also be associated with overweight and obesity [1-12]. Although there are different subtypes of abuse, like physical, sexual and mental abuse, most of the research regarding the association between abuse and weight focuses on overweight adults with a history of childhood-sexual abuse [1,2,8,9,11]. Three different mechanisms have been hypothesized to explain this association. First, it has been suggested that sexually abused individuals may adopt ineffective or immature coping styles, such as distorted eating behavior, to cope with the perceived abuse. Since maturation of coping styles [9,13,14], may be compromised by physical or mental problems [13,15], it has been hypothesized that individuals who were abused during childhood or adolescence may adopt such ineffective or immature coping styles, which may lead to overweight or even obesity. Second, overweight in itself may be used as a defense mechanism, since overweight individuals are thought to attract less attention, thereby supposedly reducing the likelihood of being abused [2,8,11,16]. However, it is less likely that becoming overweight is used as a defense mechanism against mental abuse. Literature shows that levels of (weight-related) emotional victimization are higher among obese individuals [17], thereby suggesting that obesity may cause mental abuse. Third, research shows elevated levels of hypothalamic-pituitary-adrenal (HPA) axis activity in abused children, this increased HPA activity (together with the related increased cortisol levels), has been shown to be associated with overweight and obesity among adolescents [18-21].

Although exact prevalences are unknown, research estimates that physical and mental abuse are more common among adolescents than sexual abuse, with over 100,000 victims each year in the Netherlands alone [22-24]. When physical and mental abuse as well as sexual abuse are associated with overweight and obesity, the number of overweight individuals in need of psychological support could well be dramatically higher than is currently estimated. This would have implications for the current organization and operationalization of psychological support within the treatment of overweight and obesity. Therefore, insight into the prevalence of these subtypes of abuse and their association with overweight is essential.

Felitti et al. (1993) found that significant weight gain tends to start shortly after the abuse [2]. Therefore, it is hypothesized that the association between abuse and overweight is already prevalent in adolescents. There is however very little research into this association among adolescents [3,6,25]. Two studies focusing on sexual abuse [3,25], investigated the association between abuse during childhood and overweight among young adults. A third study, included multiple subtypes of abuse [6]. In contrast, the current study focuses on being abused in childhood and becoming overweight during adolescence. When overweight as a consequence of abuse is already prevalent in adolescence, early detection and treatment of abuse might prevent adolescents from developing obesity and other long-term social, emotional and physical problems in adulthood.

The aims of the current study were 1) to investigate the prevalence of physical, sexual and mental abuse among adolescents, and 2) to examine the associations of physical, sexual and mental abuse with BMI status of adolescents.

## Methods

The current study used data from a cross-sectional survey (E-MOVO) which was originally designed to monitor health, well-being and lifestyle of adolescents. Data were gathered among second and fourth grade secondary school students aged 13–16 in the catchment area of six collaborating Community Health Services in the eastern part of the Netherlands in 2003 and 2007. During one classroom session of approximately 50 minutes, adolescents completed a classroom-based internet administered questionnaire. This questionnaire was constructed on the basis of several existing instruments used by Community Health Services and other health institutes [26]. The questionnaire contained 100 questions, on items like demographics, school, physical health, psychological health, well-being, lifestyle and social networks [27]. Though not formally validated, the questions were based on standardized questions constructed by experts in the field of concern [26]. All responses to the survey were anonymous and self-reported. Further details concerning the design of the E-MOVO study have been published elsewhere [27,28]. Both the ethics committee of TNO-PG (Dutch Institute For Applied Physical Sciences – Department Of Healthy Living) and the Dutch National Ethics Board (Central Committee on Research involving Human Subjects) concluded that formal approval by a medical ethical committee was not necessary as adolescents only had to complete an anonymous questionnaire once, which is in accordance with the guidelines laid down in the Declaration of Helsinki.

Schools with educational levels varying from lower secondary professional education (VMBO) to pre-university education (VWO) were invited to participate, schools providing education for children with special needs such as learning difficulties or mental disability were excluded as the questionnaire was not found suitable for these children.

Data were gathered by means of two separate cross-sectional surveys; one in 2003 and one in 2007. Both years, all schools in the eastern part of the Netherlands were invited to participate. In total, 77,270 adolescents completed the questionnaire either in 2003 (n = 35,107) or in 2007 (n = 42,163). Respondents with missing values on self-reported height and/or body weight were excluded (n = 20,458), as were those with a BMI lower than 8 or higher than 45 (n = 65). This resulted in a study population of 51,856 adolescents, 50,420 (97%) of whom completed the question on physical abuse, 50,173 (97%) completed the question on sexual abuse, and 50,225 (97%) completed the question on mental abuse.

### Body mass index status

Based on self-reported height and body weight their BMI was calculated. BMI was then used to classify all respondents into one of the following weight categories; underweight, normal weight, overweight or obesity. Underweight was defined by gender specific cut off points, based on a Dutch reference population [29]. Overweight and obesity were defined by international age and gender specific cut off points [30]. Remaining participants were classified as normal weight.

### Abuse

Physical, sexual and mental abuse were measured by asking the following questions: ‘Have you ever been physically abused such as kicked, beaten or tied down? Have you ever had any sexual experience without your own consent? Have you ever been mentally abused such as often picked upon, jelled at or depreciated?’ [26]. The questions on physical and mental abuse, could be answered on a four-point scale: ‘yes’, ‘currently not, but I was abused in the past’, ‘no’, and ‘I do not want to answer this question’. The question on sexual abuse could be answered on a three-point scale: ‘yes’, ‘no’, and ‘I do not want to answer this question’. Adolescents who did not want to answer a specific abuse question (1422 did not answer the physical abuse question, these numbers were 1669 and 1622 for the sexual and mental abuse question), were marked as ‘missing’ and not included in the analyses. In the final analyses on physical abuse included data of 48,998 adolescents, analysis on sexual and mental abuse included data of 48,504 and 48,603 adolescents respectively. Abuse variables were dichotomized into the following two categories; ‘yes or currently not, but I was abused in the past’ and ‘no’. Abused adolescents were also asked to indicate by whom (peer or adult) and in what setting (at home, at school, in the neighborhood, elsewhere) they had been abused.

### Covariates

Covariates included gender, ethnicity, educational level and parental communication. Ethnicity was dichotomized into Dutch and non-Dutch. Adolescents were categorized as non-Dutch if one or both of their parents was not born in the Netherlands. Educational level, a measure of socioeconomic status, was dichotomized into vocational education (lower secondary professional education) and higher secondary education (higher general secondary education and pre-university education). Parental communication was measured by asking adolescents whether they were able to have a proper talk with their parents (yes, reasonably, no).

### Data analyses

Statistical analyses were performed using the Statistical Package for Social Sciences (SPSS) Version 17.0. Descriptive statistics were conducted to get an overview of the demographic characteristics of the study population as well as the prevalence of the BMI categories and abuse subtypes. T-tests and Chi-square tests were used to identify differences in demographics, BMI status or abuse subtypes between subgroups of the study population (i.e., boys vs. girls and vocational vs. higher secondary educational level).

To examine the associations between every subtype of abuse and BMI status separately, three General Estimation Equation (GEE) analyses were performed. GEE analyses were performed to account for clustering of the students within schools [31]. First crude analyses were conducted thereafter adjusted regression models were performed, which adjusted for gender, educational level, ethnicity, parental communication, as well as the remaining two subtypes of abuse. Results were considered significant if p < 0.05.

Effect modification was checked for gender, educational level, and ethnicity. In case of a significant interaction (p<0.10) between the potential effect modifier and abuse, the analyses were stratified for the variable of concern.

## Results

**Figure 1:**
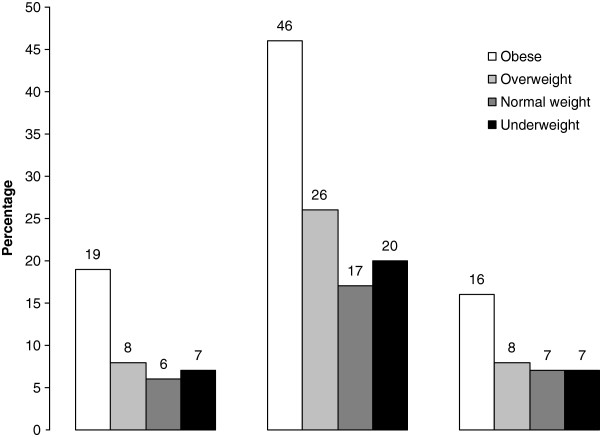
Percentages of abuse for different BMI categories.

The mean age of the adolescents was 14.4 (1.2) years (Table 1). Forty-nine percent was male and 49% attended vocational secondary education. Of all adolescents 11% was underweight, 8% was overweight and 1% was obese. Boys were significantly more often overweight compared to girls, while girls were significantly more often underweight compared to boys. Adolescents attending vocational secondary education were more often overweight compared to those attending higher secondary education. In total 6% of the adolescents was ever physically abused, 7% was ever sexually abused and 18% was ever mentally abused. These prevalences increased with an increasing BMI status, thus 8% and 19% of the overweight and obese adolescents was ever physically abused, 8% and 16% was ever sexually abused and 26% and 46% was ever mentally abused (Figure 1). For underweight adolescents, these percentages were 7%, 7% and 20% respectively. Both sexual and mental abuse were significantly more often reported by girls compared to boys. Physical, sexual and mental abuse were perceived significantly more often among adolescents attending a vocational education than among higher secondary school students (Table 1).

**Table 1:** Characteristics (in %) of the study population

	**Total (n = 51856)**	**Boys (n = 25326)**	**Girls (n = 26529)**	**Vocational education (n = 25426)**	**Higher secondary education (n = 26408)**
Mean age (SD) *	14.4 (1.2)	14.5 ( 1.3)	14.4 (1.2)	14.6 (1.2)	14.3 (1.3)
**Gender (boys)**^†^	49	-	-	50	48
**Educational level**^††^					
Vocational	49	50	48	-	-
Higher secondary education	51	50	52	-	-
**Ethnicity**^†^					
Dutch	86	86	86	84	88
Non-Dutch	14	15	14	16	12
Weight status *					
Underweight	11	9	13	10	12
Normal weight	80	81	80	79	82
Overweight	8	9	6	10	6
Obesity	1	1	1	1	1
**Physically abused**^†^	6	6	6	7	5
Sexually abused *	7	5	8	8	5
Mentally abused *	18	16	20	20	16
Good parental communication *	73	77	69	72	75

* Significant difference between boys and girls and between vocational and higher secondary education (p < 0.05).

^†^ Significant difference only between vocational and higher secondary education (p < 0.05).

^††^ Significant difference only between boys and girls (p < 0.05).

Four percent of all adolescents had perceived two subtypes of abuse, and 1% of the adolescents reported being physically, mentally and sexually abused. Being a victim of multiple forms of abuse was significantly more common among obese adolescents (51%) than among their normal weight peers (23%). Thirty-one percent of the physically abused students reported to be abused by peers at school and 31% reported being abused by an adult at home. The sexually abused adolescents most often reported (20%) that they were abused by peers somewhere else than at home, at school or in the neighborhood. Finally, 67% of the adolescents that had been mentally abused reported to have been abused by peers at school.

Within the total study population, crude analyses showed that all subtypes of abuse were significantly associated with obesity (Table 2). Although the associations between abuse and overweight were weaker, they were still significant. Only physical and mental abuse were associated with underweight. Obese adolescents were approximately four times more likely to be mentally abused compared to normal weight adolescents. Also, obese adolescents were about four times more likely to have been physically abused and three times more likely to have been sexually abused compared to their normal weight peers. However, though still significant, the adjusted model showed considerably smaller ORs for these associations (respectively 1.50 and 1.79). In these adjusted models, there was a J-shaped association with significant associations of mental abuse with underweight, overweight and obesity.

**Table 2:** Associations between weight status and abuse (physical abuse, sexual abuse and mental abuse)*

	**Crude model**		**Adjusted model**^**†**^	
	**OR**	**95% CI**	**OR**	**95% CI**
**Physically abused**				
Underweight	*1.17*	*1.02 – 1.34*	*1.21*	*1.04 – 1.40*
Normal weight	1.00	-	1.00	-
Overweight	*1.39*	*1.23 – 1.58*	1.02	0.89 – 1.18
Obesity	*3.90*	*3.04 – 5.01*	*1.50*	*1.15 – 1.96*
**Sexually abused**				
Underweight	0.96	0.85 – 1.09	0.87	0.76 – 1.00
Normal weight	1.00	-	1.00	-
Overweight	*1.15*	*1.00 – 1.31*	1.01	0.87 – 1.18
Obesity	*2.62*	*2.04 – 3.35*	*1.79*	*1.37 – 2.33*
**Mentally abused**				
Underweight	*1.16*	*1.07 – 1.25*	*1.12*	*1.03 – 1.21*
Normal weight	1.00	-	1.00	-
Overweight	*1.65*	*1.54 – 1.77*	*1.72*	*1.60 – 1.85*
Obesity	*4.02*	*3.36 – 4.79*	*3.67*	*3.02 – 4.47*

* Underweight n=47396, overweight n=45653, obesity n=42199, *Italic* figures indicate significant results p<.05.

^**†**^ Adjusted for gender, ethnicity, educational level, parental communication and the remaining subtypes of abuse.

The adjusted models in Table 3 and Table 4 showed differences in the association between abuse and BMI status for gender and educational level. In boys, mental abuse was significantly associated with underweight while in girls physical and sexual abuse were significantly associated with underweight (Table 3). In both boys and girls, mental abuse was associated with overweight and obesity, while physical and sexual abuse were associated with obesity only in boys. Mental abuse was shown to be associated with overweight and obesity in both educational levels (Table 4), while sexual abuse was associated only with obesity in both educational groups. In addition, physical and mental abuse were associated with underweight in only the vocational school students.

**Table 3:** Associations between weight status and abuse (physical abuse, sexual abuse and mental abuse) stratified by gender*

	**Boys**				**Girls**			
	**Crude model**		**Adjusted model**^**†**^		**Crude model**		**Adjusted model**^**†**^	
	**OR**	**95% CI**	**OR**	**95% CI**	**OR**	**95% CI**	**OR**	**95% CI**
**Physically abused**								
Underweight	*1.28*	*1.06 – 1.54*	1.14	0.93 – 1.40	1.10	0.92 – 1.32	*1.26*	*1.03 – 1.53*
Normal weight	1.00	-	1.00	-	1.00	-	1.00	-
Overweight	*1.33*	*1.12 – 1.57*	1.06	1.04 – 1.50	*1.51*	*1.24 – 1.84*	1.00	0.82 – 1.23
Obesity	*4.49*	*3.37 – 5.99*	*1.77*	*1.29 – 2.42*	*3.06*	*2.02 – 4.61*	1.19	0.72 – 1.96
**Sexually abused**								
Underweight	1.08	0.88 – 1.33	0.99	0.79 – 1.26	*0.84*	*0.73 – 0.98*	*0.83*	*0.71 – 0.97*
Normal weight	1.00	-	1.00	-	1.00	-	1.00	-
Overweight	*1.25*	*1.05 – 1.49*	1.07	0.88 – 1.29	1.17	0.99 – 1.40	0.95	0.77 – 1.16
Obesity	*3.82*	*2.78 – 5.25*	*2.49*	*1.82 – 3.42*	*1.85*	*1.18 – 2.90*	1.11	0.66 – 1.87
**Mentally abused**								
Underweight	*1.37*	*1.23 – 1.53*	*1.35*	*1.20 – 1.53*	1.00	0.91 – 1.11	0.99	0.89 – 1.10
Normal weight	1.00	-	1.00	-	1.00	-	1.00	-
Overweight	*1.51*	*1.35 – 1.68*	*1.51*	*1.35 – 1.70*	*1.98*	*1.78 – 2.21*	*1.97*	*1.75 – 2.21*
Obesity	*3.70*	*2.97 – 4.60*	*2.98*	*2.32 – 3.83*	*5.27*	*4.08 – 6.80*	*5.07*	*3.84 – 6.70*

* Boys; underweight n=22756, overweight n=22714, obesity n=20757. Girls; underweight n=24640, overweight n=22939, obesity n=21442, *Italic* figures indicate significant result p< .05.

^**†**^ Adjusted for ethnicity, educational level, parental communication and the remaining subtypes of abuse

**Table 4:** Associations between weight status and abuse (physical abuse, sexual abuse and mental abuse) stratified by educational level*

	**Vocational secondary education**				**Higher secondary education**			
	**Crude model**		**Adjusted model**^**†**^		**Crude model**		**Adjusted model**^**†**^	
	**OR**	**95% CI**	**OR**	**95% CI**	**OR**	**95% CI**	**OR**	**95% CI**
**Physically abused**								
Underweight	*1.23*	*1.03 – 1.45*	*1.23*	*1.02 – 1.49*	1.12	0.94 – 1.35	1.17	0.96 – 1.42
Normal weight	1.00	-	1.00	-	1.00	-	1.00	-
Overweight	*1.22*	*1.04 – 1.43*	1.04	0.87 – 1.25	*1.69*	*1.35 – 2.11*	1.02	0.80 – 1.30
Obesity	*3.38*	*2.55 – 4.49*	*1.60*	*1.17 – 2.19*	*4.60*	*3.00 – 7.06*	1.27	0.79 – 2.04
**Sexually abused**								
Underweight	0.98	0.83 – 1.16	0.89	0.74 – 1.07	0.95	0.79 – 1.15	0.85	0.70 – 1.04
Normal weight	1.00	-	1.00	-	1.00	-	1.00	-
Overweight	1.05	0.90 – 1.23	1.12	0.85 – 1.21	1.25	0.97 – 1.59	1.02	0.77 – 1.34
Obesity	*2.24*	*1.73 – 2.90*	*1.69*	*1.28 – 2.22*	*2.94*	*1.73 – 5.01*	*2.14*	*1.25 – 3.69*
**Mentally abused**								
Underweight	*1.23*	*1.11 – 1.37*	*1.15*	*1.02 – 1.30*	*1.10*	*1.00 – 1.21*	1.09	0.99 – 1.20
Normal weight	1.00	-	1.00	-	1.00	-	1.00	-
Overweight	*1.42*	*1.28 – 1.57*	*1.47*	*1.32 – 1.65*	*2.06*	*1.84 – 2.31*	*2.13*	*1.87 – 2.41*
Obesity	*3.85*	*3.11 – 4.76*	*3.70*	*2.90 – 4.72*	*4.18*	*3.08 – 5.66*	*3.57*	*2.58 – 4.93*

* Vocational secondary school; underweight n=22661, overweight n=22460, obesity n=20369. Higher secondary education; underweight n=24716, overweight n=23176, obesity n=21812, *Italic* figures indicate significant result p<.05.

^**†**^ Adjusted for gender, ethnicity, parental communication and the remaining subtypes of abuse.

## Discussion

The aims of the current study were 1) to investigate the prevalence of physical, sexual and mental abuse among adolescents, and 2) to examine the associations of physical, sexual and mental abuse with BMI status of adolescents.

Results showed that one out of five adolescents reported mental abuse, while one out of ten reported physical or sexual abuse. These prevalences are in line with expectations based on previous conducted research [22,23]. All subtypes of abuse were significantly more common among underweight, overweight and obese adolescents compared to their normal weight peers. The adjusted GEE analyses showed significant associations of mental abuse with underweight, overweight and obesity. Physical and sexual abuse were significantly associated with obesity in boys and vocational school students, and with underweight in girls.

In sum, our findings confirm that abuse is associated with underweight, overweight and obesity among adolescents. Suggesting that the hypothesized mechanisms (i.e., distorted eating behavior as an ineffective coping response, developing overweight as a defense mechanism, and elevated HPA-axis activity) through which abuse might cause an aberrant BMI status in adults apply to adolescents as well. However, due to the cross-sectional design of this study, we were not able to test these mechanisms. Hence, intermediate-analyses within longitudinal research are needed to explore the accuracy of these mechanisms. In addition to overeating, future research should also consider physical inactivity and sedentary behavior as potential responses to abuse. Abused individuals generally show signs of isolation from the community [17,32,33] as a way to protect themselves from potential abusers. However, such behavior may cause a decrease in physical activity and an increase in sedentary behavior [34] which in turn may contribute to the development of overweight.

Especially in case of physical and mental abuse, the relation with overweight may even be reciprocal. Because of their weight, overweight or obese adolescents are more often victimized, either overt or relational [17,35]. Longitudinal data are needed to examine the direction of the relationship between abuse and overweight.

Current adjusted regression results also showed that physical and sexual abuse were significantly associated with obesity in boys, but not in girls. These findings do not agree with earlier finding showing significant associations of physical and sexual abuse with overweight in women [1,2,8,10,11]. This discrepancy with our findings may be due to the age difference between the study populations. Fuemmeler and colleagues (2009) hypothesized that, unlike adult women, adolescent girls perceive a strong social pressure to be thin which might overrule the need to start overeating as a coping response towards the perceived abuse [3]. Since these are the first studies investigating the effects of different forms of abuse on overweight among adolescents, further research is needed to confirm these findings.

Contrary to findings among higher secondary school students, physical abuse was significantly associated with obesity in vocational school students. Although scientific proof is still lacking, Finkelstein et al. (2007) suggested that adolescents with a lower socioeconomic status have fewer psychological resources (e.g., optimism) to properly cope with adverse events [36]. This might trigger these individuals more easily to adopt ineffective coping style such as overeating, compared to their higher educated peers. More extensive research on the psychological resources and (lifestyle based) coping methods in response to abuse among this subpopulation is desirable.

Finally, mental abuse showed to be associated with underweight in boys and vocational school students (in the adjusted models), while physical and sexual abuse was associated with underweight in girls. This is in line with previous studies that showed distorted eating behaviour in abuse victims [6,7,37,38], which can lead to overweight or underweight and even anorexia nervosa, especially in girls [38-40].

### Strengths and limitations

This study is distinctive in the age-group of concern as well as the fact that we were able to include different types of abuse. Another strength of this study is the size of the study population. However, some limitations should be noted. First, all data were self-reported by adolescents, therefore it is likely that the prevalence of the different subtypes of abuse are underestimated. However, since adolescents have been told that all answers were strictly confidential, it is hypothesized that the current study provides a good estimation of the extent to which adolescents perceive themselves to be abused. Besides, previous research indicates that when using self-reported height and weight data, the number of adolescents classified as overweight and obese are most likely to be underestimated [41,42]. This was confirmed by yearly data, including weight and height of children aged 0 till 19, collected by the youth health care institute in the Netherlands. This institute reported that 18% of all 14-year olds was overweight (including obese) and 4% was obese [43], compared to 8% and 1% in the current study. Hence, the association between BMI status and abuse might be even larger than estimated in the current study. Second, the current study population might not be entirely representative for the total Dutch population of adolescents since there were slight differences in the questionnaires that were used by the different Community Health Services and non-response was not registered consequently by all Community Health Services. Besides, as in all voluntary based research, non-response might not be random. It could very well be that adolescents with certain specific characteristics did not (entirely) complete the questionnaire, leading to either an under- or overestimation of the current findings. Since it was not possible to conduct a non-response analysis, neither the demographics of the non-responders nor the possible differences between responders and non-responders concerning these demographics could be tested. Finally, one of the answering options was ‘I do not want to answer this question’ to the questions on the different subtypes of abuse. Since it can only be speculated whether these adolescents were abused or not, these adolescents were excluded from the current analyses. Students who did not want to answer the abuse questions had a significant higher weight status compared to students who answered these questions by ‘yes’ or ‘no’. However, the association of abuse with overweight and obesity did not differ between these sub-groups (results not shown).

## Conclusions

Mental abuse was associated with underweight, overweight and obesity, and reported by almost half of the obese adolescents, while physical and sexual abuse were reported by almost 1 out of 5 obese adolescents. Longitudinal analyses are recommended to provide insight into the causality of the association between abuse and BMI status. Furthermore, the mechanisms explaining this association should be further investigated. Especially, the role of unhealthy lifestyles such as physical inactivity, sedentary behaviour and dietary behaviour within the association are worthwhile to exploring.

The associations found between abuse and BMI status may have implications for current prevention and treatment measures. Health care professionals working in the field of health promotion should at least be aware of the high prevalence of abuse among adolescents with and aberrant BMI status. Moreover, and possibly ‘because of’, previous research showed high prevalence rates of suicidal thoughts among obese adolescents [44]. In case of severe psychological co-morbidity, it seems plausible that such adolescents benefit more from specialized psychological or welfare care, than from a strong focus on their lifestyle habits. Hence, a proper screening before inclusion within a lifestyle program is advised, especially since underlying psychological problems may be an important determinant of drop out [8].

## Competing interests

No conflict of financial or non-financial interest was declared by any of the authors.

## Authors’ contribution

J. V., K.I.P. and W. J. E. B were responsible for designing, analyzing, writing and editing the manuscript. H.B.H.M performed part of the analysis. H.B.H.M also was responsible for and provided insight in the data that were used in the present study. All authors read and approved the final manuscript.

## Pre-publication history

The pre-publication history for this paper can be accessed here:

http://www.biomedcentral.com/1471-2458/12/840/prepub
